# Rapid Access Addiction Medicine Clinics for People With Problematic Opioid Use

**DOI:** 10.1001/jamanetworkopen.2023.44528

**Published:** 2023-11-22

**Authors:** Kim Corace, Kednapa Thavorn, Kelly Suschinsky, Melanie Willows, Pamela Leece, Meldon Kahan, Larry Nijmeh, Natalie Aubin, Michael Roach, Gord Garner, Refik Saskin, Eliane Kim, Danielle Rice, Sheena Taha, Gary Garber, Brian Hutton

**Affiliations:** 1 Substance Use and Concurrent Disorders Program, Royal Ottawa Mental Health Centre, Ottawa, Ontario, Canada; 2 Faculty of Medicine, University of Ottawa, Ottawa, Ontario, Canada; 3 University of Ottawa Institute of Mental Health Research at The Royal, Ottawa, Ontario, Canada; 4 Ottawa Hospital Research Institute, Ottawa, Ontario, Canada; 5 School of Epidemiology and Public Health, Faculty of Medicine, University of Ottawa, Ottawa, Ontario, Canada; 6 Institute for Clinical Evaluative Sciences, Toronto, Ontario, Canada; 7 Department of Family and Community Medicine, University of Toronto, Toronto, Ontario, Canada; 8 Substance Use Service, Women’s College Hospital, Toronto, Ontario, Canada; 9 Public Health Ontario, Toronto, Ontario, Canada; 10 Dalla Lana School of Public Health, University of Toronto, Toronto, Ontario, Canada; 11 Lakeridge Health, Oshawa, Ontario, Canada; 12 Department of Family Medicine, Queens University, Kingston, Ontario, Canada; 13 Health Sciences North, Sudbury, Ontario, Canada; 14 School of Northern and Rural Health, Laurentian University, Sudbury, Ontario, Canada; 15 Community Addictions Peer Support Association, Ottawa, Ontario, Canada; 16 Department of Psychiatry and Behavioral Neurosciences, McMaster University, Hamilton, Ontario, Canada; 17 St Joseph’s Healthcare Hamilton, Hamilton, Ontario, Canada; 18 Canadian Centre on Substance Use and Addiction, Ottawa, Ontario, Canada

## Abstract

**Importance:**

New approaches are needed to provide care for individuals with problematic opioid use (POU). Rapid access addiction medicine (RAAM) clinics offer a flexible, low-barrier, rapid access care model for this population.

**Objective:**

To assess the associations of RAAM clinics with emergency department (ED) visits, hospitalizations, and mortality for people with POU.

**Design, Setting, and Participants:**

A retrospective cohort study involving a matched control group was performed using health administrative data from Ontario, Canada. Anonymized data from 4 Ontario RAAM clinics (cities of Ottawa, Toronto, Oshawa, and Sudbury) were linked with health administrative data. Analyses were performed on a cohort of individuals who received care at participating RAAM clinics and geographically matched controls who did not receive care at a RAAM clinic. All visits occurred between October 2, 2017, and October 30, 2019, and data analyses were completed in spring 2023. A propensity score–matching approach was used to balance confounding factors between groups, with adjustment for covariates that remained imbalanced after matching.

**Exposures:**

Individuals who initiated care through the RAAM model (including assessment, pharmacotherapy, brief counseling, harm reduction, triage to appropriate level of care, navigation to community services and primary care, and related care) were compared with individuals who did not receive care through the RAAM model.

**Main Outcomes and Measures:**

The primary outcome was a composite measure of ED visits for any reason, hospitalization for any reason, and all-cause mortality (all measured up to 30 days after index date). Outcomes up to 90 days after index date, as well as outcomes looking at opioid-related ED visits and hospitalizations, were also assessed.

**Results:**

In analyses of the sample of 876 patients formed using propensity score matching, 440 in the RAAM group (mean [SD] age, 36.5 [12.6] years; 276 [62.7%] male) and 436 in the control group (mean [SD] age, 36.8 [13.8] years; 258 [59.2%] male), the pooled odds ratio (OR) for the primary, 30-day composite outcome of all-cause ED visit, hospitalization, or mortality favored the RAAM model (OR, 0.68; 95% CI, 0.50-0.92). Analysis of the same outcome for opioid-related reasons only also favored the RAAM intervention (OR, 0.47; 95% CI, 0.29-0.76). Findings for the individual events of hospitalization, ED visit, and mortality at both 30-day and 90-day follow-up also favored the RAAM model, with comparisons reaching statistical significance in most cases.

**Conclusions and Relevance:**

In this cohort study of individuals with POU, RAAM clinics were associated with reductions in ED visits, hospitalizations, and mortality. These findings provide valuable evidence toward a broadened adoption of the RAAM model in other regions of North America and beyond.

## Key Points

**Question:**

Are rapid access addiction medicine (RAAM) clinics associated with improved clinical outcomes for people with problematic opioid use (POU)?

**Findings:**

In this cohort study of 876 individuals, important reductions in the frequency of hospitalization, emergency department visits, and death were associated with receipt of care for POU at a RAAM clinic.

**Meaning:**

The findings of this study suggest that RAAM clinics are associated with improved clinical outcomes for people with POU and can provide effective, evidence-based care while reducing wait times and improving continuity of care.

## Teaser

This cohort study of people with problematic opioid use examines the effectiveness of rapid access addiction medicine clinics on emergency department visits, hospital admissions, and mortality.

## Introduction

The world is in an opioid crisis. Nearly 90 000 deaths from opioid-related toxicity occurred worldwide in 2019, and nearly 60 million people used opioids for nonmedical purposes in 2021.^1^ The COVID-19 pandemic exacerbated the opioid crisis,^2^ particularly in North America, where deaths, hospitalizations, emergency department (ED) visits, and emergency medical service responses for opioid-related toxicity increased.^1,3,4^

Despite ongoing efforts to mitigate the opioid crisis, services for people using opioids remain fragmented, wait times are long, and access to care is limited.^5,6^ Access to evidence-based pharmacotherapy (ie, opioid agonist therapy [OAT]) for problematic opioid use (POU) and opioid use disorder remains a gap in many countries, including the US and Canada.^7,8^ Because of these gaps, as well as limited availability of clinical support and follow-up through primary care and a lack of 24-7 care options, persons with POU often present to EDs to access care. Easy care access is associated with improved retention in treatment for POU^9,10^; however, EDs often lack appropriate and easy-to-access referral destinations for these individuals.^11^ Thus, flexible, low-barrier (ie, without an appointment or referral), rapid access care models are needed.

To address this need in Ontario, Canada, rapid access addiction medicine (RAAM) clinics began implementation in 2015 through the META:PHI (Mentoring, Education, and Clinical Tools for Addiction: Partners in Health Integration) Network, a pilot project to support the rollout of 7 RAAM clinics. These clinics are flexible (eg, walk-in hours), low-barrier, rapid access care models staffed by multidisciplinary teams of addiction medicine physicians, nurse practitioners, nurses, and social workers or outreach workers.^12^ Patients receive specialist care at RAAM clinics and are connected to primary care for long-term follow-up, with the option to return to a RAAM clinic at any time if needed. RAAM clinicians provide ongoing support to primary care practitioners by being available for reassessments, consultations, and substance use disorder (SUD) management guidance. Patients move throughout the care pathway, with communication between practitioners in the different settings. With the success of the META:PHI project, the Ontario Ministry of Health expanded funding to include 60 RAAM clinics. META:PHI supports quality standards and best practices, education and clinical guidance, mentorship, and policy.^12,13^ In some countries, such as the US, care for individuals with SUD involves separation of medical treatment of SUD complications, primary care treatments, and behavioral health therapies; care from addiction specialists is uncommon, and efforts from primary care services can be unsuccessful in establishing SUD care and corresponding medications. RAAM clinics help overcome siloed care to maximize support for individuals with POU.

The presence of RAAM clinics in Canada is growing,^14^ and there are also signs of growth of the RAAM model in the US.^15,16,17,18,19,20,21^ Preliminary evidence suggests that RAAM clinics are effective at reducing follow-up ED visits for problematic alcohol use (PAU) or alcohol use disorder (AUD), reducing substance use in individuals with PAU and POU, improving mental health symptoms, and retaining individuals in care.^22,23^ There is currently limited literature on the clinical benefits and effectiveness of the RAAM model. We sought to assess the effectiveness of RAAM clinics on ED visits, hospital admissions, and mortality in individuals with POU.

## Methods

### Study Design

A protocol was prepared a priori,^24^ and the final report was prepared in consideration of the Strengthening the Reporting of Observational Studies in Epidemiology (STROBE) and Reporting of Studies Conducted Using Observational Routinely-Collected Data (RECORD) reporting guidelines.^25^ eFigure 1 in Supplement 1 presents the study design based on the Structured Template and Reporting Tool for Real World Evidence (StaRT-RWE) reporting guidance for community-setting evidence studies.^26^ Through linkage of anonymized clinical and administrative databases, we performed a retrospective cohort study involving 4 RAAM clinics from Canadian cities within the province of Ontario. To reduce residual confounding among measured covariates when estimating the effects of exposure to a RAAM clinic, attendees from RAAM clinics (ie, treatment group) were matched with similar individuals who did not attend RAAM (control group) in the same geographic catchment region using the propensity score methods. Any covariates still imbalanced after matching were adjusted for in data analyses. The protocol for this study was approved by the research ethics boards at The Ottawa Hospital and The Royal Ottawa Healthcare Group (Ottawa), Women’s College Hospital (Toronto), and Health Sciences North (Sudbury). The proposal was deemed to be a program evaluation project and exempt from research ethics board review at Lakeridge Health (Oshawa).

### Study Setting and Participants

RAAM clinics were selected for participation based on the following criteria: operational for at least 6 months; at least 2 days per week with walk-in hours; estimated monthly number of new patients of at least 15; availability of a range of expertise, including a physician or nurse practitioner with addiction medicine expertise, a nurse, and/or social worker or counselor; and providing service to individuals with opioid use, alcohol use, and/or other substance use problems. Centers were required to provide the core components of the RAAM model, including (1) rapid assessment (for substance use health, mental health, physical health, and other immediate needs); (2) pharmacotherapy for POU, alcohol use, and other substance use (eg, OAT, such as buprenorphine-naloxone); (3) brief counseling for POU, alcohol use, and other substance use; (4) triage to appropriate care level (ie, inpatient vs outpatient; navigation to community services); (5) harm reduction (ie, education, supplies, and naloxone kits); (6) connection with primary care; and (7) connection to community treatment and resources (ie, community treatment beds and psychosocial counseling) for support. On the first clinic visit, individuals are assessed first by the clinic’s counselor, nurse, or social worker. Individuals are then seen by a physician or nurse practitioner, who prescribes OAT if clinically indicated. All RAAM clinics prescribe buprenorphine-naloxone and, through partnerships, provide individuals with methadone as appropriate. The RAAM model integrates pharmacologic treatments, psychosocial and behavioral treatments, addiction medicine, and primary care into 1 team to address substance use issues.^12,13^ Four Ontario-based RAAM clinics met these criteria and were selected for the study: the Royal Ottawa Mental Health Centre (Ottawa, Ontario, Canada), Lakeridge Health (Oshawa, Ontario, Canada), Health Sciences North (Sudbury, Ontario, Canada), and Women’s College Hospital (Toronto, Ontario, Canada). Additional details regarding each RAAM clinic are available in eTable 1 in Supplement 1.

Our population included individuals 15 years or older who were eligible to receive care for POU (including opioid use disorder). Outcomes were compared between those who presented for POU to a RAAM clinic (treatment group) vs those who did not present to a RAAM clinic and used other pathways of care, such as EDs (control group). Individuals required a valid unique identifier, had known sex and dates of birth and death (where applicable), and were residents of Ontario, Canada, at the time of index visit (ie, first clinic visit in the RAAM group; in the control group, we used the established approach of assigning index dates randomly by incidence density sampling from the distribution of index dates in the RAAM cohort).^27,28,29^ Individuals who presented 1 or more times were eligible, with no requirements related to contact frequency. All observations were excluded for individuals who appeared in both the RAAM and control groups, as were observations for individuals who were identified as candidate controls who presented to more than 1 RAAM clinic.

### Data Sources

This study was conducted at the Institute for Clinical Evaluative Sciences (ICES), an independent, nonprofit research institute whose legal status under Ontario’s health information privacy law allows it to collect and analyze health care and demographic data, without consent, for health system evaluation and improvement. Secure access to these data is governed by policies and procedures that are approved by the Information and Privacy Commissioner of Ontario. Each RAAM clinic provided ICES a listing of individuals who attended the clinic for POU during their study period, which varied among clinics in relation to their opening date (Ottawa: April 3, 2018, to October 29, 2019; Toronto: October 2, 2017, to September 23, 2019; Oshawa: April 1, 2018, to October 31, 2019; and Sudbury: January 8, 2018, to October 30, 2019). Data were checked for outliers; missing data were labeled as “no known address” for postal codes or “none” for health card numbers. Missing data were not imputed. Attendee lists were linked to administrative databases using a unique and encrypted provincial health insurance number for each clinic attendee. A summary of the administrative data sources maintained by ICES and used in this study is provided in eTable 2 in Supplement 1.

### Outcome Measures

The primary outcome was a composite measure of 30-day events of clinical relevance that included ED visits for any reason, hospitalizations for any reason, and all-cause death. Secondary 30-day outcomes included the individual components of the primary outcome; a composite outcome of events, including ED visits and hospitalizations for opioid-related reasons and all-cause death; ED visits for opioid-related reasons; and hospitalizations for opioid-related reasons. In addition to the 30-day composite and individual outcome measures, these outcomes were also assessed at 90 days. The number of days in the hospital for any reason after 90 days was also assessed. These outcomes were chosen via discussion with the research team, which included individuals with lived experience, clinicians, epidemiologists, and stakeholders (ie, META:PHI).

### Methods for Propensity Score Matching

Propensity score matching was used to form a control group whose characteristics were comparable to the RAAM groups at each center. Propensity scores were calculated as the probability of being seen at a RAAM clinic (vs the control intervention [not seen at a RAAM clinic]) using logistic regression conditional on a series of baseline covariates. Four matched cohorts were formed (1 per clinic), each consisting of pairs of individuals who attended a RAAM clinic and their matched controls who did not, the latter drawn from an eligible population from the same geographic region. Propensity score matching was performed to produce comparable sets of RAAM-exposed and RAAM-unexposed individuals, seeking to mimic the effect from a randomized clinical trial.^30^ In this study, the preintervention measures used to derive propensity scores included age and sex; Charlson Comorbidity Index; indexes of rurality, dependence, neighborhood income, deprivation, instability, and ethnic concentration; and history of comorbidities within 2 years of index visit, including history of an opioid use disorder–related hospitalization, AUD, substance use, psychotropic drug use, mood disorder, mental distress, suicide attempt, chronic lung disease, heart disease, hepatitis, cirrhosis, and the numbers of hospitalizations, ED visits, and general practitioner visits. Codes used to establish the covariates and outcomes for each patient from administrative data are described in eTables 3 to 5 in Supplement 1.

After propensity score calculation, matching was performed. The logit of the calculated propensity score for each participant (using a caliper of 0.20 times the SD of the logit of the propensity score), geographic region of related RAAM clinic, age at index date, and sex were used to match patients within the RAAM and control groups. The distributions of propensity scores at each center were assessed before and after matching to assess overlap. Standardized mean differences (SMDs) were estimated to assess differences between groups before and after matching, where SMDs greater than 0.10 were considered indicative of potentially notable differences between groups.

### Statistical Analysis

We used hierarchical modeling to adjust for any unbalanced covariates from the propensity score matching approach and compared effects of the RAAM model and control for each outcome. The model treated the intervention group (RAAM or control) as a fixed effect and clinic as a random effect. Covariates associated with an SMD greater than 0.10 after matching were included as fixed effects in all models. Bayesian information criterion was used to assess fit of models including vs omitting covariate adjustment. Comparisons were reported as odds ratios (ORs) with 95% CIs, with ORs less than 1 favoring the RAAM model. Analyses were completed in spring 2023 and were conducted through a secure ICES portal using SAS Software (SAS Institute Inc), with all patient data anonymized during linkage.

## Results

### Overview of Cohort Characteristics

**Figure 1.**
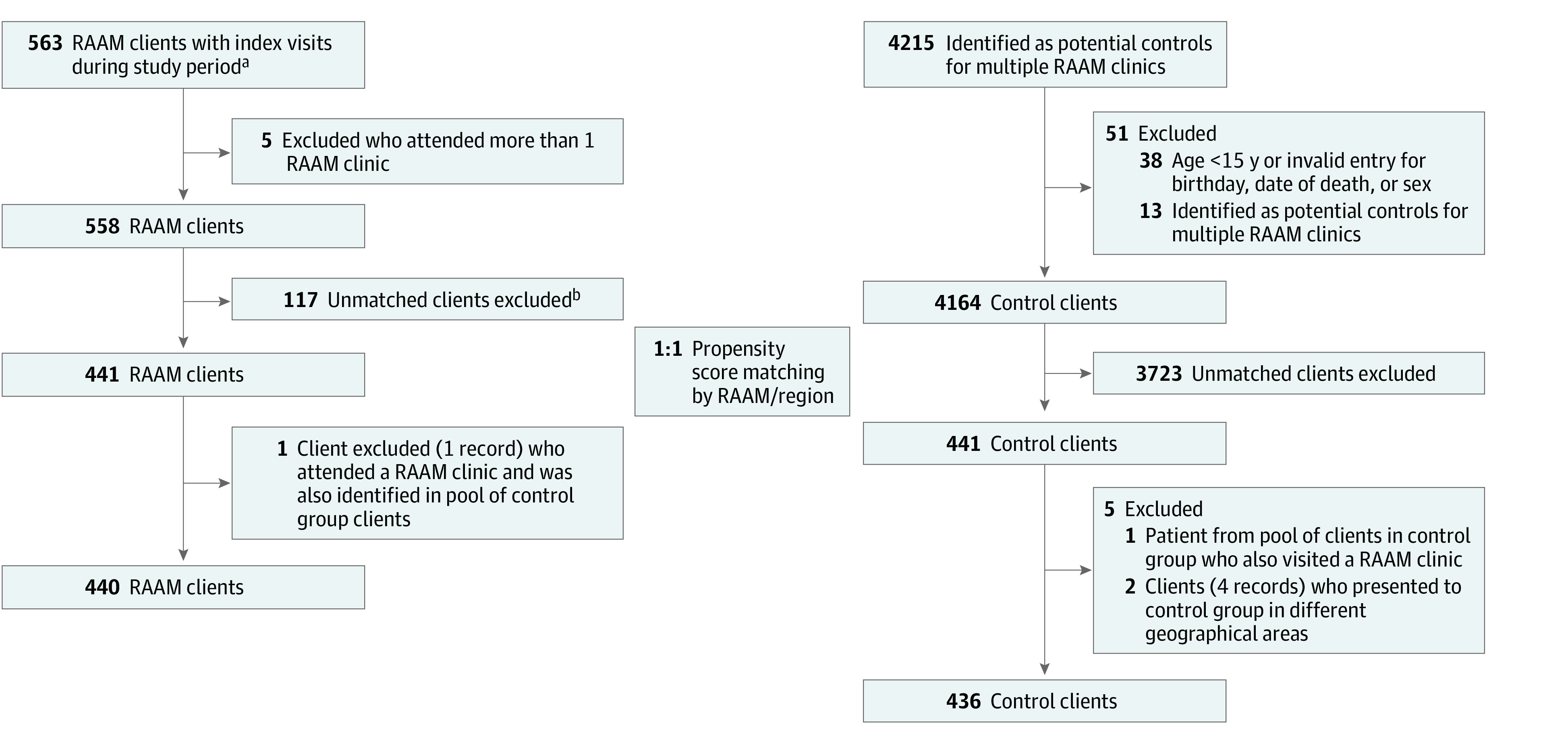
Formation of the Study Cohort and Control Group ^a^Rapid access addiction medicine (RAAM) patients were linked at the time of selection of the control population. ^b^Among the 117 unmatched RAAM patients, 110 at the Oshawa clinic could not be matched because of a low number of eligible controls.

Of the 4722 eligible individuals, 876 matched observations were made, 440 in the RAAM group (mean [SD] age, 36.5 [12.6] years; 276 [62.7%] male and 164 [37.3%] female ) and 436 in the control group (mean [SD] age, 36.9 [13.8] years; 258 [59.2%] male and 178 [40.8%] female). Figure 1 summarizes the numbers of eligible patients before and after use of 1:1 propensity score matching. Before matching, differences between groups for each location were identified based on inspection of SMDs (eTable 6 in Supplement 1). Differences between the RAAM and control groups for many measures were observed, including age, sex, comorbidity history at index date (including histories of prior hospitalization related to opioid use, AUD, SUD, mental distress, and others), Charlson Comorbidity Index, and indexes of deprivation, income, dependency, and ethnic diversity.

During the study period, 563 individuals presented to a participating RAAM clinic, whereas 4215 eligible controls presented to a corresponding ED. Before matching, 5 individuals from the RAAM group who attended more than 1 RAAM clinic were excluded, as were 51 in the control group (38 who were aged <15 years or had an invalid entry for birthday, date of death, or sex; and 13 who were identified as potential controls at multiple RAAM clinics). After 1:1 matching, 441 matched pairs (186 in Ottawa, 80 in Toronto, 127 in Oshawa, and 48 in Sudbury) were formed; 1 individual who attended a RAAM clinic in 1 city and was identified as a control in a different city was excluded from analyses (n = 2 observations), and 2 individuals were initially identified as matched controls in 2 different cities (n = 4 observations); all observations from these individuals were excluded, leaving 876 observations for analysis (440 in the RAAM group and 436 in the control group).

The Table presents the characteristics of the RAAM and control groups across centers before and after matching. The SMDs comparing baseline measures between groups were all less than 0.10, with the exception of the Charlson Comorbidity Index, indicating strong overall balance between groups. The Charlson Comorbidity Index was adjusted for in all analyses to account for this difference between groups. Density plots of the distributions of propensity scores in the exposure groups before and after matching showed strong overlap (eFigures 2-5 in Supplement 1). Because only 159 eligible controls were identified during the study period for the geographic region of the Oshawa RAAM (compared with 237 individuals who attended RAAM), only 127 matched pairs for this center could be formed. The SMDs suggested certain differences between the matched and unmatched Oshawa RAAM patients (eTable 7 in Supplement 1), with most differences suggesting greater morbidity in the RAAM group.

**Table.** Population Traits Before and After Propensity Score Matching^a^

Covariate	Before matching			After matching		
	RAAM (n = 558)	Control (n = 4164)	SMD	RAAM (n = 440)	Control (n = 436)	SMD
Age, y						
Mean (SD)	36.5 (12.8)	41.5 (15.2)	0.35	36.5 (12.6)	36.8 (13.8)	0.03
Median (IQR)	34 (27-45)	38 (29-52)	0.33	34 (27-45)	34 (27-44.5)	0.00
Sex						
Female	212 (38.0)	1491 (35.8)	0.05	164 (37.3)	178 (40.8)	0.07
Male	346 (62.0)	2673 (64.2)	0.05	276 (62.7)	258 (59.2)	0.07
Health history						
Hospitalization due to opioid use	228 (40.9)	1406 (33.8)	0.15	194 (44.1)	187 (42.9)	0.02
Alcohol disorder	147 (26.3)	1074 (25.8)	0.01	115 (26.1)	114 (26.2)	0.00
Substance use	340 (60.9)	2356 (56.6)	0.09	284 (64.6)	284 (65.1)	0.01
Psychotropic drug use	44 (7.9)	308 (7.4)	0.02	35 (8.0)	36 (8.3)	0.01
Mood disorder	142 (25.4)	957 (23.0)	0.06	120 (27.3)	113 (25.9)	0.03
Mental distress	360 (64.5)	2661 (63.9)	0.01	299 (68.0)	298 (68.4)	0.01
Suicide attempt	0	0	NA	0	0	NA
Chronic lung disease	21 (3.8)	330 (7.9)	0.18	18 (4.1)	17 (3.9)	0.01
Heart disease	40 (7.2)	647 (15.5)	0.27	35 (8.0)	37 (8.5)	0.02
Hepatitis	31 (5.6)	255 (6.1)	0.02	26 (5.9)	22 (5.0)	0.04
Cirrhosis	NR	NR	NR	NR	NR	NR
No. of hospitalizations						
Mean (SD)	1.0 (2.1)	1.5 (3.8)	0.17	1.1 (2.2)	1.0 (2.4)	0.00
Median (IQR)	0 (0-1)	0 (0-2)	0.17	0 (0-1)	0 (0-1)	0.00
No. of ED visits						
Mean (SD)	10.7 (23.2)	14.3 (30.8)	0.13	11.1 (22.7)	11.03(17.7)	0.00
Median (IQR)	5 (2-13)	7 (3-15)	0.17	6 (3-13)	6 (3-13)	0.08
No. of GP visits						
Mean (SD)	49.9 (60.1)	58.2 (67.1)	0.13	53.2 (63.9)	55.7 (64.0)	0.04
Median (IQR)	28 (14-60)	34 (13-78)	0.10	30 (15-67)	35 (14-73)	0.05
Charlson Comorbidity Index						
Mean (SD)	0.41 (1.07)	0.99 (1.82)	0.39	0.43 (1.10)	0.75 (1.53)	0.23
Median (IQR)	0 (0-0)	0 (0-1)	0.41	0 (0-0)	0 (0-1)	0.15
Ontario rurality index, mean	NR	NR	NR	NR	NR	NR
Neighborhood income quintile						
1	179 (32.1)	1788 (42.9)	0.23	164 (37.3)	160 (36.7)	0.01
2	131 (23.5)	894 (21.5)	0.05	116 (26.4)	110 (25.2)	0.03
3	102 (18.3)	585 (14.0)	0.12	69 (15.7)	71 (16.3)	0.02
4	75 (13.4)	417 (10.0)	0.11	47 (10.7)	52 (11.9)	0.04
5	NR	NR	NR	NR	NR	NR
Missing	NR	NR	NR	NR	NR	NR
Deprivation quintile						
1	100 (17.9)	623 (15.0)	0.08	80 (18.2)	79 (18.1)	0.00
2	100 (17.9)	473 (11.4)	0.19	58 (13.2)	56 (12.8)	0.01
3	95 (17.0)	563 (13.5)	0.10	77 (17.5)	65 (14.9)	0.07
4	107 (19.2)	812 (19.5)	0.01	79 (18.0)	89 (20.4)	0.06
5	145 (26.0)	1533 (36.8)	0.23	136 (30.9)	139 (31.9)	0.02
Missing	11 (2.0)	160 (3.8)	0.11	10 (2.3)	8 (1.8)	0.03
Instability quintile						
1	50 (9.0)	166 (4.0)	0.20	32 (7.3)	32 (7.3)	0.00
2	73 (13.1)	359 (8.6)	0.14	40 (9.1)	46 (10.6)	0.05
3	100 (17.9)	486 (11.7)	0.18	71 (16.1)	72 (16.5)	0.01
4	125 (22.4)	760 (18.2)	0.10	108 (24.6)	105 (24.1)	0.01
5	199 (35.7)	2233 (53.6)	0.37	179 (40.7)	173 (39.7)	0.02
Missing	11 (2.0)	160 (3.8)	0.11	10 (2.3)	8 (1.8)	0.03
Dependency quintile						
1	150 (26.9)	1129 (27.1)	0.01	119 (27.1)	113 (25.9)	0.03
2	115 (20.6)	976 (23.4)	0.07	98 (22.3)	99 (22.7)	0.01
3	111 (19.9)	640 (15.4)	0.12	77 (17.5)	78 (17.9)	0.01
4	87 (15.6)	552 (13.3)	0.07	65 (14.8)	65 (14.9)	0.00
5	84 (15.0)	707 (17.9)	0.05	71 (16.1)	73 (16.7)	0.02
Missing	11 (2.0)	160 (3.8)	0.11	10 (2.3)	8 (1.8)	0.03
Ethnic diversity quintile						
1	78 (14.0)	402 (9.6)	0.13	61 (13.9)	72 (16.5)	0.07
2	111 (19.9)	407 (9.8)	0.29	82 (18.6)	70 (16.1)	0.07
3	151 (27.1)	713 (17.1)	0.24	117 (26.6)	104 (23.9)	0.06
4	130 (23.3)	1105 (26.5)	0.07	109 (24.8)	121 (27.8)	0.07
5	77 (13.8)	1377 (33.1)	0.47	61 (13.9)	61 (14.0)	0.00
Missing	11 (2.0)	160 (3.8)	0.11	10 (2.3)	8 (1.8)	0.03

Abbreviations: ED, emergency department; GP, general practitioner; NA, not applicable; NR, not reported (because of small sample size); RAAM, rapid access addiction medicine; SMD, standardized mean difference.

^a^ Data are presented as number (percentage) unless otherwise indicated.

### Clinical Outcomes at 30 Days

**Figure 2.**
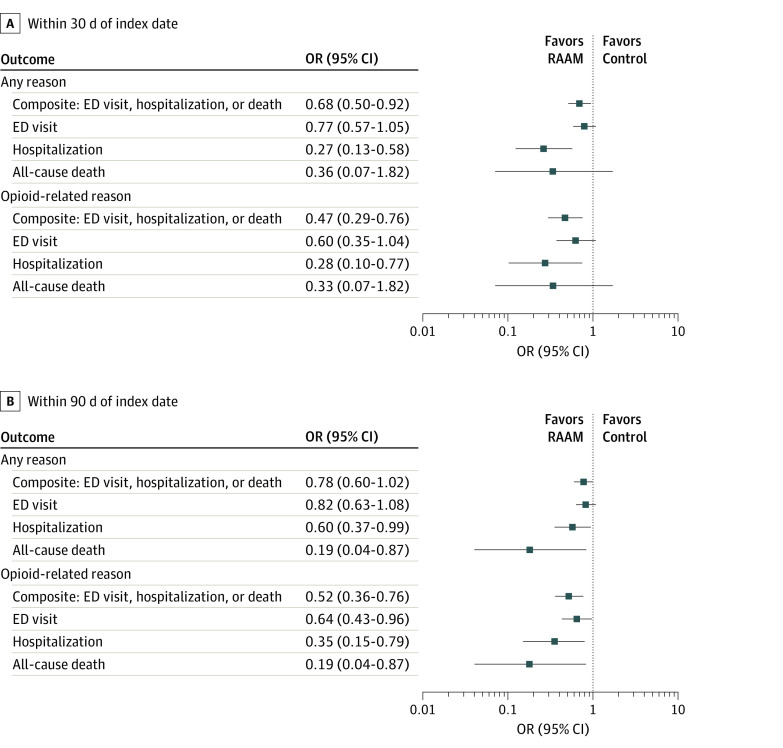
Comparison of Clinical Outcomes Within 30 Days and 90 Days of the Index Date Findings from all 30-day outcomes (A) and 90-day outcomes (B) comparing the rapid access addiction model (RAAM) model (n = 440) and control (n = 436) based on multilevel modeling analyses of the propensity score–matched sample are presented. Analyses involving outcomes that occurred for any reason, as well as for opioid-related reasons, are both presented. For all analyses, odds ratios (ORs) less than 1 favor the RAAM model. ED indicates emergency department.

Figure 2A presents the findings pertaining to 30-day outcomes. Analyses of the primary composite outcome found the RAAM model to be associated with a statistically significant reduction in the occurrence of the composite outcome of ED visits for any reason, hospitalizations for any reason, and 30-day all-cause mortality compared with the control group (OR, 0.68; 95% CI, 0.50-0.92; 100 of 440 [22.7%] RAAM vs 132 of 436 [30.3%] control). Statistically significant differences favoring the RAAM model were also noted for the outcome of hospitalizations for any reason (OR, 0.27; 95% CI, 0.13-0.58; 9 of 440 [2.1%] RAAM vs 32 of 436 [7.3%] control), whereas comparisons for all-cause ED visits and mortality numerically favored the RAAM model but did not reach statistical significance.

Analyses focused on opioid-related ED visits or hospitalizations and all-cause mortality demonstrated findings consistent with those above. For the composite measure involving all 3 events, odds were significantly reduced with the RAAM model (OR, 0.47; 95% CI, 0.29-0.76; 28 of 440 [6.4%] RAAM vs 56 of 436 [12.8%] control). The RAAM model was also associated with significantly lower odds of hospitalization (OR, 0.28; 95% CI, 0.10-0.77; unable to report group-level data because of small numbers of events) and lower odds of ED visits and overall death, although statistical significance was not reached (Figure 2A). The underlying numbers of events for all outcomes are provided in eTable 8 in Supplement 1.

### Clinical Outcomes at 90 Days

Figure 2B presents results from analyses of the same outcomes with follow-up extended to 90 days after index date, with findings demonstrating a high degree of consistency compared with those observed at 30 days. Comparison of the odds of the composite outcome of hospitalization, ED visit, and all-cause mortality favored the RAAM model but did not reach significance (OR, 0.78; 95% CI, 0.60-1.02; 185 of 440 [42.0%] RAAM vs 212 of 436 [48.6%] control), and estimates of effect for both hospitalization for any reason (OR, 0.60; 95% CI, 0.37-0.99; 29 of 440 [6.6%] RAAM vs 48 of 436 [11.0%] control) and all-cause mortality (OR, 0.19; 95% CI, 0.04-0.87; unable to report group-level data due to small numbers of events) demonstrated statistically significant differences between groups.

Analyses that focused on events related specifically to opioid use were performed. Statistically significant differences favoring the RAAM model were identified for the composite measure of hospitalization, ED visit, or all-cause mortality (OR, 0.52; 95% CI, 0.36-0.76; 52 of 440 [11.8%] RAAM vs 89 of 436 [20.4%] control), as well as for the individual components of hospitalization (OR, 0.35; 95% CI, 0.15-0.79; 8 of 440 [1.8%] RAAM vs 22 of 436 [5.0%] control) and ED visit (OR, 0.64; 95% CI, 0.43-0.96; 46 of 440 [10.4%] RAAM vs 67 of 436 [15.4%] control). The underlying numbers of events for all outcomes are provided in eTable 8 in Supplement 1.

### Length of Hospitalization

Comparisons of length of hospital stay demonstrated a mean (SD) length of stay of 11.76 (63.60) hours for the RAAM model and 19.68 (82.32) hours for the control group. The corresponding medians (IQRs) were 0 (0-0) in both groups.

## Discussion

To our knowledge, this is the first multicenter study demonstrating the clinical benefits of the RAAM model in Canada. Our findings demonstrated associations between RAAM clinic attendance and important clinical benefits related to hospitalizations, ED visits, and mortality after both 30 and 90 days (compared with those with POU who did not attend a RAAM clinic). Findings were similar when considering both all-cause and opioid-related outcomes. These results contribute to increasing evidence of the effectiveness of rapid access care models, particularly the impact on reducing acute health care use. Srivastava et al^31^ conducted a randomized clinical trial of individuals with POU or PAU residing in withdrawal management services. Individuals in the rapid intervention group (ie, those given an appointment at an addiction medicine clinic within 2 days) had fewer ED visits in the 6 months after their referral compared with individuals in the delayed intervention group (ie, those given contact information for the addiction medicine clinic). Koser et al^32^ studied individuals referred to a “bridge” clinic, which provided rapid, low-barrier access to initial treatment from a multidisciplinary team with transition to longer-term care. They reported reduced ED visits and hospitalizations among the individuals with health care use data from the 6-month period before and after referral to the bridge clinic. Our findings show reduced ED visits and hospitalizations 30 and 90 days after initial RAAM presentation, suggesting that RAAM clinics may help reduce health care use.

In our study, RAAM was also associated with mortality reduction. This finding may be partially attributable to easy access to OAT at RAAM clinics. Opioid agonist therapy is associated with reduced bloodborne illnesses, fatal overdoses, and all-cause mortality.^33,34,35^ Initiations of OAT in the ED (with subsequent transfers to community care) are associated with increased treatment retention after discharge from the ED.^20,36^ Thus, initiation of OAT in the ED followed by a referral to a RAAM clinic may contribute to improved outcomes.

Although this research demonstrated improved outcomes for individuals served by RAAM clinics, additional work is needed to examine outcomes related to quality of life and the differential impact of outcomes on marginalized populations given that gender-diverse, racialized, and minoritized communities face increased barriers to care. Evaluation of the return on investment of RAAM clinics is also needed, and we are currently performing this work.

### Limitations

This study has certain limitations of note. First, given the observational design, a causal relationship between the RAAM model and improved clinical outcomes cannot be confirmed. Second, given that certain additional confounders (eg, receipt of other services, including community-based, nonmedical withdrawal management services, counseling, and/or bed-based treatment services) could not be acquired for the control population derived from administrative data, residual confounding cannot be ruled out. Third, due to the paucity of eligible controls identified in the Oshawa region, we were unable to match all Oshawa RAAM patients; importantly, the fact that RAAM patients who were matched and included in the analyses for this region were associated with more comorbidities than the unmatched RAAM patients (and were well balanced with the control group) suggests that the effectiveness of RAAM clinics presented in this work may be conservative. Fourth, although exploring the effects of the RAAM model in different settings was of interest, limitations of sample size precluded the potential for robust analyses. Given that RAAM clinics from various geographic areas across Ontario were included in this study, these findings are generalizable to similar RAAM clinics in Ontario and potentially elsewhere in Canada. We hope to conduct prospective research to confirm these findings with inclusion of additional RAAM clinics, a larger sample size, and additional relevant outcomes, such as treatment engagement over time and use of OAT, that were not feasible within this study.

## Conclusions

The findings of this cohort study suggest that RAAM clinics are associated with important benefits to individuals with POU, including a reduced overall risk of mortality, hospitalizations, and ED visits. These findings provide evidence supporting a broadened adoption of the RAAM model and provide decision makers, practitioners, and communities with evidence urgently needed to collaboratively implement novel interventions to address opioid-related harms.

## Notes

Supplement 1. **eTable 1.** Overview of Participating RAAM Clinic Characteristics **eTable 2.** ICES Databases Overview **eTable 3.** Covariate Definitions and Codes – Patient Characteristics **eTable 4.** Codes, Identification of Hospitalizations and Emergency Department Visits Related to Opioids **eTable 5.** List of Opioid Poisoning ICD-10-CA Codes **eTable 6.** Pre-Match Distribution of Characteristics, by Center **eFigure 1.** Overview of Study Design **eFigure 2.** Propensity Score Density Plot, Pre-Match vs Post-Match for Ottawa Center **eFigure 3.** Propensity Score Density Plot, Pre-Match vs Post-Match for Toronto Center **eFigure 4.** Propensity Score Density Plot, Pre-Match vs Post-Match for Oshawa Center **eFigure 5.** Propensity Score Density Plot, Pre-Match vs Post-Match for Sudbury Center **eTable 7.** Comparison of Matched and Unmatched RAAM Clients, Oshawa Clinic **eTable 8.** Number of Events per Outcome Measure

Supplement 2.
Data Sharing Statement
