# Supplementary material for: Rapid Access Addiction Medicine Clinics for People With Problematic Opioid Use
Source: JAMA Netw Open. 2023 Nov 22;6(11):e2344528. doi: 10.1001/jamanetworkopen.2023.44528 (PMC10665968; doi:10.1001/jamanetworkopen.2023.44528)
Supplement: Supplement 1. — eTable 1. Overview of Participating RAAM Clinic Characteristics eTable 2. ICES Databases Overview eTable 3. Covariate Definitions and Codes – Patient Characteristics eTable 4. Codes, Identification of Hospitalizations and Emergency Department Visits Related to Opioids eTable 5. List of Opioid Poisoning ICD-10-CA Codes eTable 6. Pre-Match Distribution of Characteristics, by Center eFigure 1. Overview of Study Design eFigure 2. Propensity Score Density Plot, Pre-Match vs Post-Match for Ottawa Center eFigure 3. Propensity Score Density Plot, Pre-Match vs Post-Match for Toronto Center eFigure 4. Propensity Score Density Plot, Pre-Match vs Post-Match for Oshawa Center eFigure 5. Propensity Score Density Plot, Pre-Match vs Post-Match for Sudbury Center eTable 7. Comparison of Matched and Unmatched RAAM Clients, Oshawa Clinic eTable 8. Number of Events per Outcome Measure [file jamanetwopen-e2344528-s001.pdf]

Supplementary Online Content

Corace K, Thavorn K, Suschinsky K, et al. Rapid access addiction medicine clinics for people with problematic opioid use. *JAMA Netw Open*. 2023;6(11):e2344528. doi:10.1001/jamanetworkopen.2023.44528

eTable 1. Overview of Participating RAAM Clinic Characteristics

eTable 2. ICES Databases Overview

eTable 3. Covariate Definitions and Codes – Patient Characteristics

eTable 4. Codes, Identification of Hospitalizations and Emergency Department Visits Related to Opioids

eTable 5. List of Opioid Poisoning ICD-10-CA Codes

eTable 6. Pre-Match Distribution of Characteristics, by Center

eFigure 1. Overview of Study Design

eFigure 2. Propensity Score Density Plot, Pre-Match vs Post-Match for Ottawa Center

eFigure 3. Propensity Score Density Plot, Pre-Match vs Post-Match for Toronto Center

eFigure 4. Propensity Score Density Plot, Pre-Match vs Post-Match for Oshawa Center

eFigure 5. Propensity Score Density Plot, Pre-Match vs Post-Match for Sudbury Center

eTable 7. Comparison of Matched and Unmatched RAAM Clients, Oshawa Clinic

eTable 8. Number of Events per Outcome Measure

This supplementary material has been provided by the authors to give readers additional information about their work.

© 2023 Corace K et al. *JAMA Network Open.*

eTable 1. Overview of Participating RAAM Clinic Characteristics

Location and Date of Opening for Problematic Opioid Use	Clinic Setting	Clinic Location	Walk-in times	Average monthly # of all new patients	Average monthly # of new patients with opioid problems	% with opioid use; alcohol use; other substance use	Distribution of referral sources	Age distribution or average age	Gender distribution
Royal Ottawa Mental Health Centre (Ottawa)<br><br>***February 2018***	Substance Use and Concurrent Disorders Program at a mental health centre.	Urban	Tuesdays and Wednesdays 8am to 11am (Walk-in from ED for alcohol daily Mon-Friday)	52	10	17% opioids; 76% alcohol; 23% other substances	62% ED; 34% self-referral; 1% primary care; 4% primary care, community agencies	*M* age = 40 years old<br><br><=17 = 1%<br>18-25 = 15%<br>26-64 = 79%<br>65+ = 5%	M = 63%<br>F = 35%<br>Trans or nonbinary < 2%
Lakeridge Health Centre (Oshawa)<br><br>***January 2018***	Two days per week at withdrawal management services location; two days at hospital	Urban	Monday, Tuesday, Thursday, Friday from 9am-11am	40	11	41% opioids; 49% opioids and alcohol; 44% alcohol; 15% other	17% ED; 15% self-referral; 62% withdrawal management	18-25 = 11%<br>26-64 = 83%<br>65+ = 6%	M = 60%<br>F = 40%
Women's College Hospital (Toronto)<br><br>***March 2017***	Ambulatory hospital	Urban	Mondays, Tuesdays, and Thursdays 10:00–12:00	28	2	23% opioids; 63% alcohol; 14% other	13% ED; 41% self-referral; 22% primary care; 8% withdrawal management; 11% community agencies	18-25 = 9%<br>26-64 = 87%<br>65+ = 4%	unavailable
Health Sciences North (Sudbury)<br><br>***December 2015***	Withdrawal management services	Urban	Daily Monday to Friday (9-4pm)	15	NR*	20% opioids, 60% alcohol 18% Other 2% Stimulants	19% ED; 27% self-referred; 16% primary care; 19% Health Sciences North; 10% withdrawal management	<=17 = 2%<br>18-25 = 12%<br>26-64 = 70%<br>65+ = 16%	M= 57%<br>F = 43%

© 2023 Corace K et al. *JAMA Network Open.*

eTable 2. ICES Databases Overview

Database	Purpose of Use
Registered Persons Database	To identify age, sex, and location of residence
National Ambulatory Care Reporting System	To identify clinical information such as diagnoses and procedures from patient visits to hospital- and community-based ambulatory care centers
Discharge Abstract Database	To identify clinical information related to admissions to acute care hospitals, rehabilitation, chronic and day surgery institutions
Ontario Marginalization Index	To identify information in terms of four dimensions that include *dependency* (which identifies neighbourhoods that have high proportions of seniors, children, and adults for whom either work is uncompensated and/or they cannot work as a result of disability); *ethnic diversity* (which is a measurement of area-level concentrations of recent immigrants to Canada and/or individuals who belong to a “visible minority” group as defined by Statistics Canada); *material deprivation* (which connects conceptually to poverty and socio-economic position and associated with indicators of low income, educational attainment, housing quality and characteristics of family housing); and *residential instability* (which measures a neighbourhood’s cohesiveness and volatility, and which also relates to indicators of housing types and population density)
Ontario Health Insurance Plan	To gather information regarding outpatient services covered by provincial insurance plan

© 2023 Corace K et al. *JAMA Network Open.*

eTable 3. Covariate Definitions and Codes – Patient Characteristics

Variable/Condition and Definition	ICD10 Codes
History of hospital admission due to opioid use	F11, T40.0, T40.1, T40.2, T40.3, T40.4, T40.6, T45.0
History of alcohol use disorder	K70, F10
History of substance use	F10, F11, F12, F13, F14, F15, F16, F19, R78, T40
History of psychotropic drug use	T43
History of mood disorder	F3
History of mental distress	F1, F2, F3, F40, F41, F42, F43, F44, F45, F46, F47, F48, F50, F6, F90, F99
History of chronic lung disease	ICES Chronic Obstructive Lung Disease cohort
History of heart disease	I10, I11, I12, I13, I14, I15, I16, I20, I21, I22, I23, I24, I25, I33, I38, I39, I60, I61, I63, I64, I65, I66, I67, I68, I69
History of hepatitis	B17, B18
History of cirrhosis	K71.7, K74.3, K74.4, K74.5, K74.6
History of suicide attempt	T14.91

eTable 4. Codes, Identification of Hospitalizations and Emergency Department Visits Related to Opioids

ICD10 Code	Variable/Condition and Definition
Mental and behavioural disorders due to use of opioids, acute intoxication	F11.0
Mental and behavioural disorders due to use of opioids, harmful use	F11.1
Mental and behavioural disorders due to use of opioids, dependence syndrome	F11.2
Mental and behavioural disorders due to use of opioids, withdrawal state	F11.3
Mental and behavioural disorders due to use of opioids, withdrawal state with delirium	F11.4
Mental and behavioural disorders due to use of opioids, psychotic disorder	F11.5
Mental and behavioural disorders due to use of opioids, amnesic syndrome	F11.6
Mental and behavioural disorders due to use of opioids, residual and late-onset psychotic disorder	F11.7
Mental and behavioural disorders due to use of opioids, other mental and behavioural disorders	F11.8
Mental and behavioural disorders due to use of opioids, unspecified mental and behavioural disorder	F11.9
Drugs, medicaments and biological substances causing adverse effects in therapeutic use: opioids and related analgesics	Y45.0

© 2023 Corace K et al. *JAMA Network Open.*

eTable 5. List of Opioid Poisoning ICD-10-CA Codes

Drug name	ICD-10-CA code
Laudanum	T40.0
Opium alkaloids (total)	T40.0
Opium alkaloids (total) — Standardized powdered	T40.0
Opium alkaloids (total) — Tincture (camphorated)	T40.0
Papaveretum	T40.0
Paregoric	T40.0
Diacetylmorphine	T40.1
Diamorphine	T40.1
Heroin	T40.1
Acemorphan	T40.2
Antitussive NEC — Codeine mixture	T40.2
Antitussive NEC — Opiate	T40.2
Codeine	T40.2
Dihydrocodeine	T40.2
Dihydrocodeinone	T40.2
Dihydrohydroxycodeinone	T40.2
Dihydromorphinone	T40.2
Drocode	T40.2
Ethylmorphine	T40.2
Hydrocodone	T40.2
Hydromorphone	T40.2
Methylmorphine	T40.2
Morfin	T40.2
Morphine	T40.2
Nicomorphine	T40.2
Opioid NEC	T40.2
Oxycodone	T40.2
Oxymorphone	T40.2
Amidone	T40.3
Methadone	T40.3
Alfentanil	T40.4
Alphaprodine	T40.4
Anileridine	T40.4
Bezitramide	T40.4
Buprenorphine	T40.4
Butorphanol	T40.4
Dextromoramide	T40.4

© 2023 Corace K et al. *JAMA Network Open.*

Drug name	ICD-10-CA code
Dextropropoxyphene	T40.4
Dipipanone	T40.4
Eptazocine	T40.4
Ethoheptazine	T40.4
Fentanyl	T40.4
Isonipecaine	T40.4
Ketobemidone	T40.4
Levopropoxyphene	T40.4
Levorphanol	T40.4
Meperidine	T40.4
Nalbuphine	T40.4
Narcotic NEC — Synthetic NEC	T40.4
Pentazocine	T40.4
Pethidine	T40.4
Phenazocine	T40.4
Phenoperidine	T40.4
Piritramide	T40.4
Profadol	T40.4
Propoxyphene	T40.4
Sufentanil	T40.4
Tilidine	T40.4
Tramadol	T40.4
Analgesic NEC — Narcotic NEC	T40.6
Analgesic NEC — Narcotic NEC — Combination	T40.6
Analgesic NEC — Narcotic NEC — Obstetric	T40.6
Narcotic NEC	T40.6
Opiate NEC	T40.6

Note: NEC denotes not elsewhere classified

© 2023 Corace K et al. *JAMA Network Open.*

eTable 6. Pre-Match Distribution of Characteristics, by Center

Characteristic	Summary measure/ categories	Ottawa			Toronto			Oshawa			Sudbury		
		RAAM (N=196)	Control (N=685)	SMD	RAAM (N=80)	Control (N=2,763)	SMD	RAAM (N=237)	Control (N=159)	SMD	RAAM (N=50)	Control (N=570)	SMD
**Age (years)**	Mean/SD	33.01 (11.06)	42.02 (16.66)	**0.64**	39.03 (11.86)	41.95 (14.84)	**0.22**	38.24 (13.42)	40.64 (14.49)	**0.17**	37.98 (14.68)	38.54 (15.33)	0.04
	Med/IQR	31 (25-38)	38 (29-54)	**0.59**	37 (29 - 48)	39 (30 - 53)	**0.17**	34 (28 - 48)	39 (31 - 49)	**0.18**	34.5 (27 - 50)	34.5 (27 - 49)	0.01
**Sex**	% female	62 (31.6%)	283 (41.3%)	**0.20**	40 (50.0%)	946 (34.2%)	**0.32**	90 (38.0%)	59 (37.1%)	0.02	22 (44.0%)	207 (36.3%)	**0.16**
	% male	134 (68.4%)	402 (58.7%)	**0.20**	40 (50.0%)	1,817 (65.8%)	**0.32**	147 (62.0%)	100 (62.9%)	0.02	28 (56.0%)	363 (63.7%)	**0.16**
**History of hospital admission from opioid use**	# (%) yes	84 (42.9%)	229 (33.4%)	**0.19**	45 (56.3%)	969 (35.1%)	**0.44**	84 (35.4%)	62 (39.0%)	0.07	20 (40.0%)	151 (26.5%)	**0.29**
**History of alcohol disorder**	# (%) yes	61 (31.1%)	146 (21.3%)	**0.22**	18 (22.5%)	796 (28.8%)	**0.14**	58 (24.5%)	34 (21.4%)	0.07	11 (22.0%)	105 (18.4%)	0.09
**History of substance use**	# (%) yes	132 (67.3%)	371 (54.2%)	**0.27**	54 (67.5%)	1,628 (58.9%)	**0.18**	127 (53.6%)	97 (61.0%)	**0.15**	32 (64.0%)	270 (47.4%)	**0.34**
**History of psychotropic drug use**	# (%) yes	13 (6.6%)	56 (8.2%)	0.06	6 (7.5%)	196 (7.09%)	0.02	24 (10.1%)	19 (12.0%)	0.06	NR*	NR*	**0.11**
**History of mood disorder**	# (%) yes	48 (24.5%)	129 (18.8%)	**0.14**	21 (26.3%)	646 (23.4%)	0.07	64 (27.0%)	60 (37.7%)	**0.23**	10 (20.0%)	126 (22.1%)	0.05
**History of mental distress**	# (%) yes	136 (69.4%)	425 (62.0%)	**0.16**	55 (68.8%)	1,793 (64.9%)	0.08	141 (59.5%)	115 (72.3%)	**0.27**	33 (66.0%)	338 (59.3%)	**0.14**
**History of suicide attempt**	# (%) yes	0 (0%)	0 (0%)	NA	0 (0.00%)	0 (0.00%)	0.00	0 (0.00%)	0 (0.00%)	0.00	0 (0.00%)	0 (0.00%)	0.00
**History of chronic lung disease**	# (%) yes	NR*	NR*	NR*	NR*	NR*	NR*	10 (4.22%)	7 (4.40%)	0.01	NR*	NR*	NR*
**History of heart disease**	# (%) yes	8 (4.1%)	115 (16.8%)	**0.42**	8 (10.0%)	452 (16.4%)	**0.19**	17 (7.17%)	17 (10.7%)	**0.12**	7 (14.0%)	63 (11.1%)	0.09
**History of hepatitis**	# (%) yes	19 (9.7%)	45 (6.6%)	**0.11**	NR*	NR*	NR*	6 (2.53%)	7 (4.40%)	0.10	NR*	NR*	NR*
**History of cirrhosis**	# (%) yes	NR*	NR*	NR*	NR*	NR*	NR*	0 (0.00%)	0 (0.00%)	0.00	0 (0.00%)	NR*	NR*
**Number of hospitalizations**	Mean ± SD	1.04 (2.39)	1.39 (2.67)	**0.14**	1.16 (1.86)	1.58 (4.27)	**0.13**	0.78 (1.95)	0.95 (2.05)	0.09	1.10 (1.56)	1.26 (2.20)	0.08
	Med (IQR)	0 (0-1)	1 (0-2)	**0.22**	0.5 (0 - 2)	0 (0 - 2)	0.04	0 (0 - 1)	0 (0 - 1)	0.08	0 (0 - 2)	0 (0 - 2)	0.01
**Number of ED visits**	Mean ± SD	11.62 (26.76)	10.82 (16.21)	0.04	13.16 (24.26)	15.72 (35.96)	0.08	9.94 (22.08)	11.68 (14.55)	0.09	7.34 (6.34)	11.96 (16.71)	**0.37**
	Med (IQR)	6 (3-14)	6 (3-13)	0.03	6 (3 - 13.5)	7 (3 - 16)	0.08	4 (2 - 10)	8 (4 - 16)	**0.37**	6 (2 - 11)	7 (3 - 13)	**0.22**
**Number of GP visits**	Mean ± SD	59.36 (74.80)	59.51 (79.98)	0.00	60.81 (60.52)	63.77 (68.06)	0.05	41.10 (47.87)	50.58 (46.29)	**0.20**	33.40 (27.99)	31.25 (36.25)	0.07
	Med (IQR)	29 (13-80)	34 (13-69)	0.00	38 (17.5 - 85.5)	38 (15 - 91)	0.04	25 (13 - 50)	37 (17 - 71)	**0.32**	26 (11 - 46)	19(8 - 42)	**0.23**
**Charlson Score**	Mean ± SD	0.33 (0.72)	0.74 (1.42)	**0.37**	0.57 (1.44)	1.12 (1.98)	**0.32**	0.23 (0.68)	0.83 (1.65)	**0.48**	0.76 (1.71)	0.69 (1.40)	0.05
	Med (IQR)	0 (0-0)	0 (0-1)	**0.32**	0 (0 - 1)	0 (0 - 1)	**0.39**	0 (0 - 0)	0 (0 - 1)	**0.40**	0 (0 - 1)	0 (0 - 1)	0.07
**Ontario Rurality Index**	% < 40	13 (6.6%)	38 (5.5%)	0.05	*1 – 5	*9 - 13	0.08	9 (3.80%)	0 (0.00%)	**0.28**	6 (12.0%)	NR*	NR*
**Neighborhood income quantile**	1	59 (30.1%)	252 (36.8%)	**0.14**	24 (30.0%)	1,181 (42.7%)	**0.27**	78 (32.9%)	90 (56.6%)	**0.49**	19 (38.0%)	267 (46.8%)	**0.18**
	2	52 (26.5%)	168 (24.5%)	0.05	19 (23.8%)	574 (20.8%)	0.07	50 (21.1%)	38 (23.9%)	0.07	12 (24.0%)	119 (20.9%)	0.07
	3	40 (20.4%)	89 (13.0%)	**0.20**	13 (16.3%)	410 (14.8%)	0.04	44 (18.57%)	19 (12.0%)	**0.18**	NR*	NR*	0.01

© 2023 Corace K et al. *JAMA Network Open.*

Characteristic	Summary measure/ categories	Ottawa			Toronto			Oshawa			Sudbury		
		RAAM (N=196)	Control (N=685)	SMD	RAAM (N=80)	Control (N=2,763)	SMD	RAAM (N=237)	Control (N=159)	SMD	RAAM (N=50)	Control (N=570)	SMD
	4	19 (9.7%)	92 (13.4%)	**0.12**	NR*	NR*	**0.22**	NR*	NR*	**0.33**	10 (20.0%)	73 (12.8%)	**0.20**
	5	NR*	NR*	NR*	10 (12.5%)	276 (9.99%)	0.08	NR*	NR*	**0.38**	NR*	NR*	0.03
	Missing	NR*	NR*	NR*	NR*	NR*	NR*	0 (0.00%)	0 (0.00%)	0.00	0 (0.00%)	NR*	NR*
**Deprivation Quintile**	1	53 (27.0%)	165 (24.1%)	0.07	21 (26.3%)	434 (15.7%)	**0.26**	NR*	NR*	NR*	NR*	NR*	NR*
	2	33 (16.8%)	119 (17.4%)	0.01	NR*	NR*	NR*	NR*	NR*	NR*	9 (18.0%)	47 (8.25%)	**0.29**
	3	33 (16.8%)	84 (12.3%)	**0.13**	18 (22.5%)	388 (14.0%)	0.22	38 (16.0%)	23 (14.5%)	0.04	NR*	NR*	0.05
	4	27 (13.8%)	116 (16.9%)	0.09	13 (16.3%)	519 (18.8%)	0.07	59 (24.9%)	31 (19.5%)	**0.13**	10 (20.0%)	150 (26.3%)	0.15
	5	41 (20.9%)	161 (23.5%)	0.06	21 (26.3%)	1,052 (38.1%)	**0.26**	63 (26.6%)	85 (53.5%)	**0.57**	21 (42.0%)	236 (41.4%)	0.01
	Missing	9 (4.6%)	40 (5.8%)	0.06	NR*	NR*	NR*	0 (0.00%)	0 (0.00%)	0.00	NR*	NR*	NR*
**Instability Quintile**	1	14 (7.1%)	61 (8.9%)	0.06	NR*	NR*	NR*	32 (13.5%)	10 (6.29%)	**0.24**	NR*	NR*	NR*
	2	24 (12.2%)	80 (11.7%)	0.02	NR*	NR*	NR*	37 (15.6%)	11 (6.92%)	**0.28**	NR*	NR*	NR*
	3	29 (14.8%)	75 (10.9%)	**0.12**	7 (8.75%)	271 (9.81%)	0.04	54 (22.8%)	30 (18.9%)	0.10	10 (20.0%)	111 (19.5%)	0.01
	4	45 (23.0%)	111 (16.2%)	**0.17**	17 (21.3%)	518 (18.8%)	0.06	54 (22.8%)	50 (31.5%)	**0.20**	10 (20.0%)	85 (14.9%)	**0.13**
	5	75 (38.3%)	318 (46.4%)	**0.17**	46 (57.5%)	1,651 (59.8%)	0.05	60 (25.32%)	58 (36.5%)	**0.24**	20 (40.0%)	211 (37.0%)	0.06
	Missing	9 (4.6%)	40 (5.8%)	0.06	NR*	NR*	NR*	0 (0.00%)	0 (0.00%)	0.00	NR*	NR*	NR*
**Dependency Quintile**	1	61 (31.1%)	207 (30.2%)	0.02	32 (40.0%)	876 (31.7%)	**0.17**	51 (21.5%)	28 (17.6%)	0.10	NR*	NR*	NR*
	2	38 (19.4%)	130 (19.0%)	0.01	16 (20.0%)	707 (25.6%)	**0.13**	49 (20.7%)	34 (21.4%)	0.02	14 (28.0%)	109 (19.1%)	**0.21**
	3	30 (15.3%)	100 (14.6%)	0.02	17 (21.3%)	441 (16.0%)	**0.14**	54 (22.8%)	28 (17.6%)	**0.13**	11 (22.0%)	73 (12.8%)	**0.24**
	4	24 (12.2%)	92 (13.4%)	0.04	NR*	NR*	NR*	48 (20.3%)	32 (20.1%)	0.00	11 (22.0%)	140 (24.6%)	0.06
	5	34 (17.3%)	116 (16.9%)	0.01	9 (11.3%)	371 (13.4%)	0.07	35 (14.8%)	37 (23.3%)	**0.22**	7 (14.0%)	185 (32.5%)	**0.45**
	Missing	9 (4.6%)	40 (5.8%)	0.06	NR*	NR*	NR*	0 (0.00%)	0 (0.00%)	0.00	NR*	NR*	NR*
**Ethnic Diversity Quintile**	1	27 (13.8%)	80 (11.7%)	0.06	NR*	NR*	NR*	NR*	NR*	NR*	17 (34.0%)	265 (46.5%)	**0.26**
	2	33 (16.8%)	112 (16.4%)	0.01	NR*	NR*	NR*	56 (23.6%)	31 (19.5%)	0.10	17 (34.0%)	151 (26.5%)	**0.16**
	3	52 (26.5%)	142 (20.7%)	**0.14**	20 (25.0%)	451 (16.3%)	**0.22**	71 (30.0%)	40 (25.2%)	**0.11**	9 (18.0%)	83 (14.6%)	0.09
	4	45 (23.0%)	198 (28.9%)	**0.14**	26 (32.5%)	824 (29.8%)	0.06	55 (23.2%)	59 (37.1%)	**0.31**	NR*	NR*	NR*
	5	30 (15.3%)	113 (16.5%)	0.03	23 (28.8%)	1,258 (45.5%)	**0.35**	NR*	NR*	NR*	NR*	NR*	NR*
	Missing	9 (4.6%)	40 (5.8%)	0.06	NR*	NR*	NR*	0 (0.00%)	0 (0.00%)	0.00	NR*	NR*	NR*

Covariates/categories where an SMD >0.10 remained after matching are shown in bold/underlined font.

NR* denotes not reported due to small cell count.

© 2023 Corace K et al. *JAMA Network Open.*

eTable 7. Comparison of Matched and Unmatched RAAM Clients, Oshawa Clinic

Characteristics	Summary measure/ categories	RAAM matched (N=127)	RAAM unmatched (N=110)	SMD
Age at index date	Mean (SD)	38.94 (13.40)	37.44 (13.47)	**0.11**
	Median (IQR)	35 (28 - 49)	34 (28 - 47)	**0.11**
Sex on RPDB, n(%)	Female	46 (36.22%)	44 (40%)	0.08
	Male	81 (63.78%)	66 (60%)	0.08
History of hospital admission due to opioid use, n(%)	# (%) yes	55 (43.31%)	29 (26.36%)	**0.36**
History of alcohol disorder, n(%)	# (%) yes	30 (23.62%)	28 (25.45%)	0.04
History of substance use, n(%)	# (%) yes	79 (62.2%)	48 (43.64%)	**0.38**
History of psychotropic drug use, n(%)	# (%) yes	14 (11.02%)	10 (9.09%)	0.06
History of mood disorder, n(%)	# (%) yes	45 (35.43%)	19 (17.27%)	**0.42**
History of mental distress, n(%)	# (%) yes	87 (68.5%)	54 (49.09%)	0.4
History of suicide, n(%)	# (%) yes	0(0.00%)	0 (0.00%)	.
History of chronic lung disease, n(%)	# (%) yes	*5 – 9	*1 – 5	NR*
History of heart disease, n(%)	# (%) yes	*12 – 16	*1 – 5	NR*
History of hepatitis, n(%)	# (%) yes	*4 – 8	*1 – 5	NR*
History of cirrhosis, n(%)	# (%) yes	0 (0.00%)	0 (0.00%)	.
Number of hospitalizations 5yrs prior to index	Mean (SD)	0.97 (2.30)	0.55 (1.42)	**0.22**
	Median (IQR)	0 (0 - 1)	0 (0 - 1)	**0.24**
Number of ED visits 5yr prior to index	Mean (SD)	10.72 (17.72)	9.04 (26.28)	0.07
	Median (IQR)	5 (3 - 12)	3 (2 - 8)	**0.35**
Number of GP visits 5yrs prior to index	Mean (SD)	48.12 (57.15)	33.00 (32.61)	**0.32**
	Median (IQR)	27 (13 - 55)	21 (12 - 41)	**0.21**
Charlson Score	Mean (SD)	0.27 (0.67)	0.18 (0.73)	**0.13**
	Median (IQR)	0 (0 - 0)	0 (0 - 0)	**0.37**
Ontario Rurality Index, n(%)	No	127(100%)	101 (91.82%)	**0.42**
	Yes	0 (0.00%)	9 (8.18%)	**0.42**
Nearest Census Based Neighbourhood Income Quintile(within CMA/CA), n(%)	1	64 (50.39%)	14 (12.73%)	**0.89**
	2	35 (27.56%)	15 (13.64%)	**0.35**
	3	15 (11.81%)	29 (26.36%)	**0.38**
	4	7 (5.51%)	26 (23.64%)	**0.53**
	5	6 (4.72%)	26 (23.64%)	**0.56**
Deprivation Quintile, n(%)	1	9 (7.09%)	16 (14.55%)	**0.24**
	2	12 (9.45%)	40 (36.36%)	**0.68**
	3	20 (15.75%)	18 (16.36%)	0.02
	4	30 (23.62%)	29 (26.36%)	0.06
	5	56 (44.09%)	7 (6.36%)	**0.96**
Instability Quintile, n(%)	1	*14 - 18	*14 – 18	NR*
	2	*1 - 5	*32 – 36	NR*
	3	26 (20.47%)	28 (25.45%)	**0.12**
	4	40 (31.5%)	14 (12.73%)	**0.46**
	5	42 (33.07%)	18 (16.36%)	**0.39**
Dependency Quintile, n(%)	1	22 (17.32%)	29 (26.36%)	**0.22**
	2	32 (25.2%)	17 (15.45%)	**0.24**
	3	22 (17.32%)	32 (29.09%)	**0.28**
	4	26 (20.47%)	22 (20%)	0.01
	5	25 (19.69%)	10 (9.09%)	**0.31**
Ethnic Concentration Quintile, n(%)	1	20 (15.75%)	12 (10.91%)	**0.14**
	2	27 (21.26%)	29 (26.36%)	**0.12**
	3	37 (29.13%)	34 (30.91%)	0.04
	4	35 (27.56%)	20 (18.18%)	**0.22**
	5	8 (6.3%)	15 (13.64%)	**0.25**

SMDs >0.10 (shown in bold / underlined font) suggest potentially important differences

NR* denotes not reported due to small cell count.

© 2023 Corace K et al. *JAMA Network Open.*

eTable 8. Number of Events per Outcome Measure

Outcome	RAAM (N=440)	Control (N=436)	Odds Ratio and 95% CI
**Outcomes at 30 days**			
Composite outcome for 30 days post index: Emergency Department visit for any diagnosis, or hospitalization for any diagnosis, or all-cause mortality	100 (22.7%)	132 (30.3%)	0.68 (0.50, 0.92)
ED visit: 30 days post index, all diagnosis	99 (22.5%)	119 (27.3%)	0.77 (0.57, 1.05)
Hospitalization: 30 days post index, all diagnosis	9 (2.05%)	32 (7.34%)	0.27 (0.13, 0.58)
All-cause mortality, 30 days post index	NR*	NR*	0.36 (0.07, 1.82)
Composite outcome for 30 days post index: opioid-related Emergency Department visit, or opioid-related hospitalization, or all-cause mortality	28 (6.36%)	56 (12.8%)	0.47 (0.29, 0.76)
Emergency Department visit: 30 days post index, opioid-related diagnosis	23 (5.23%)	36 (8.26%)	0.60 (0.35, 1.04)
Hospitalization: 30 days post index, opioid-related diagnosis	NR*	NR*	0.28 (0.10 ,0.77)
All-cause mortality, 30 days post index	NR*	NR*	0.33 (0.07, 1.82)
**Outcomes at 90 days**			
Composite outcome for 90 days post index: Emergency Department visit for any diagnosis, or hospitalization for any diagnosis, or all-cause mortality	185 (42.1%)	212 (48.6%)	0.78 (0.60 ,1.02)
Emergency Department visit: 90 days post index, any diagnosis	182 (41.4%)	202 (46.3%)	0.82 (0.63 ,1.08)
Hospitalization: 90 days post index, any diagnosis	29 (6.59%)	48 (11.0%)	0.60 (0.37, 0.99)
All-cause mortality, 90 days post index	NR*	NR*	0.19 (0.04, 0.87
Composite outcome for 90 days post index: opioid-related Emergency Department visit, or opioid-related hospitalization, or all-cause mortality	52 (11.8%)	89 (20.4%)	0.52 (0.36, 0.76)
Emergency Department visit: 90 days post index, opioid-related diagnosis	46 (10.5%)	67 (15.4%)	0.64 (0.43, 0.96)
Hospitalization: 90 days post index, opioid-related diagnosis	8 (1.82%)	22 (5.05%)	0.35 (0.15, 0.79)
All-cause mortality, 90 days post index	NR*	NR*	0.19 (0.04, 0.87)

The numbers of events in the RAAM and control groups are presented alongside the associated estimate of effect for each outcome estimated using multi-level modeling as described in the main text of this manuscript.

NR* denotes # of events not reported due to small cell size

© 2023 Corace K et al. *JAMA Network Open.*

eFigure 1. Overview of Study Design

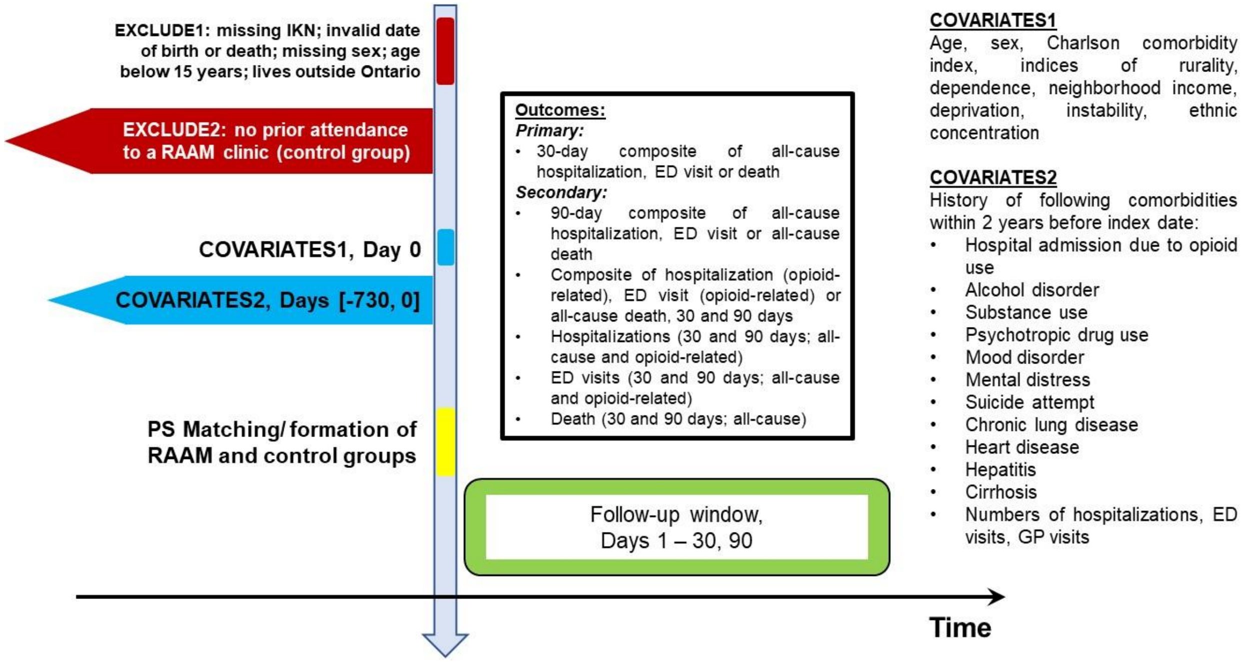

An overview of the retrospective cohort design is presented, covering the topics of patient selection, covariate measurement, outcomes of interest, study follow-up, and covariates used for propensity score matching.

Abbreviations: COVARIATES1 = covariates assessed at index date; COVARIATES2 = covariates assessed prior to index date; ED = emergency department; EXCLUDE1=exclusion criteria, part 1; EXCLUDE2 = exclusion criteria, part 2; GP = general practitioner; IKN = IC/ES key number; RAAM = rapid access addiction medicine.

© 2023 Corace K et al. *JAMA Network Open.*

eFigure 2. Propensity Score Density Plot, Pre-Match vs Post-Match for Ottawa Center

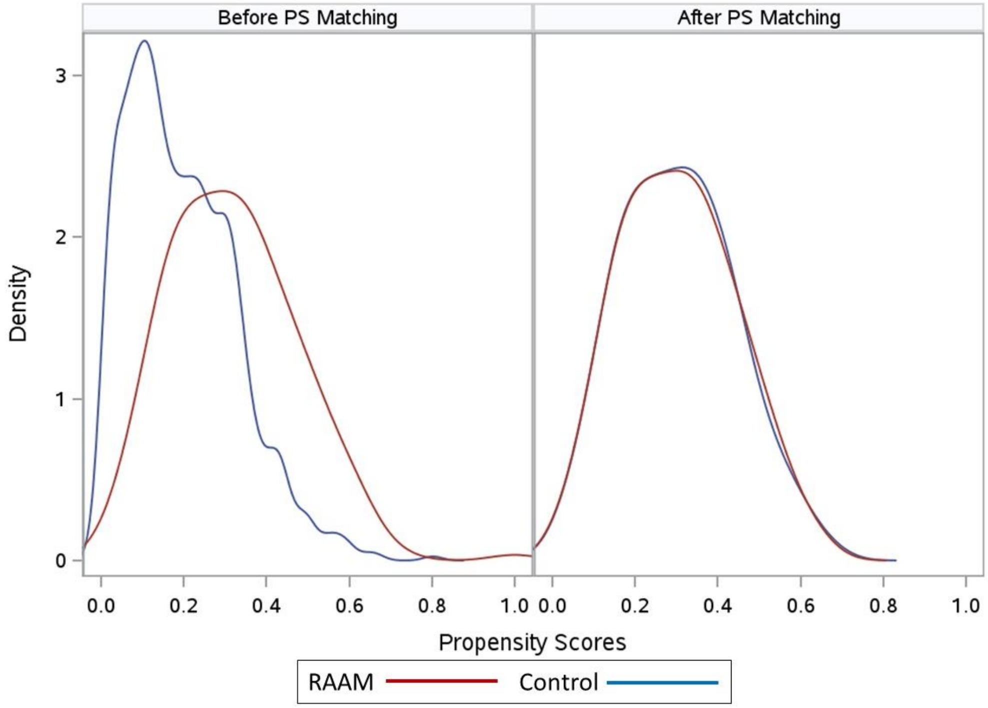

eFigure 3. Propensity Score Density Plot, Pre-Match vs Post-Match for Toronto Center

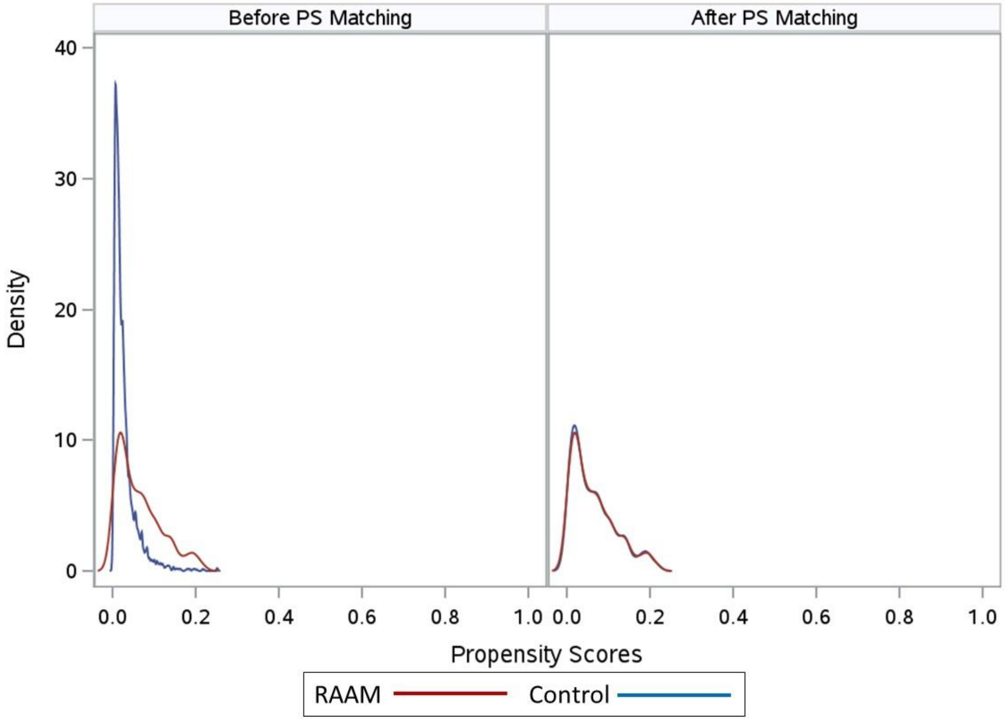

© 2023 Corace K et al. *JAMA Network Open.*

eFigure 4. Propensity Score Density Plot, Pre-Match vs Post-Match for Oshawa Center

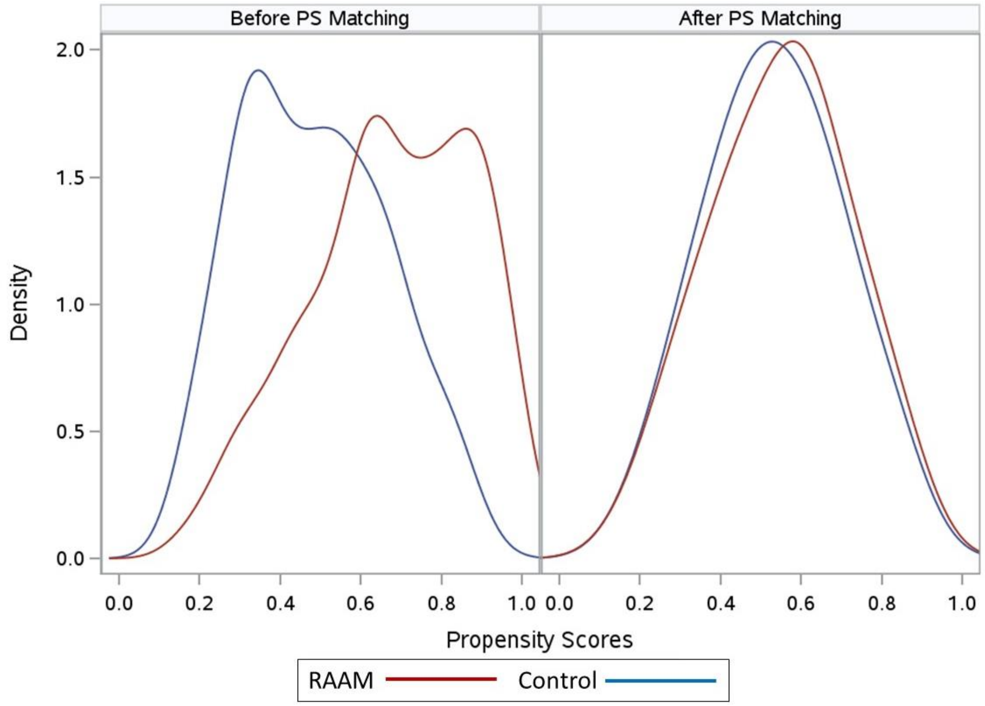

eFigure 5. Propensity Score Density Plot, Pre-Match vs Post-Match for Sudbury Center

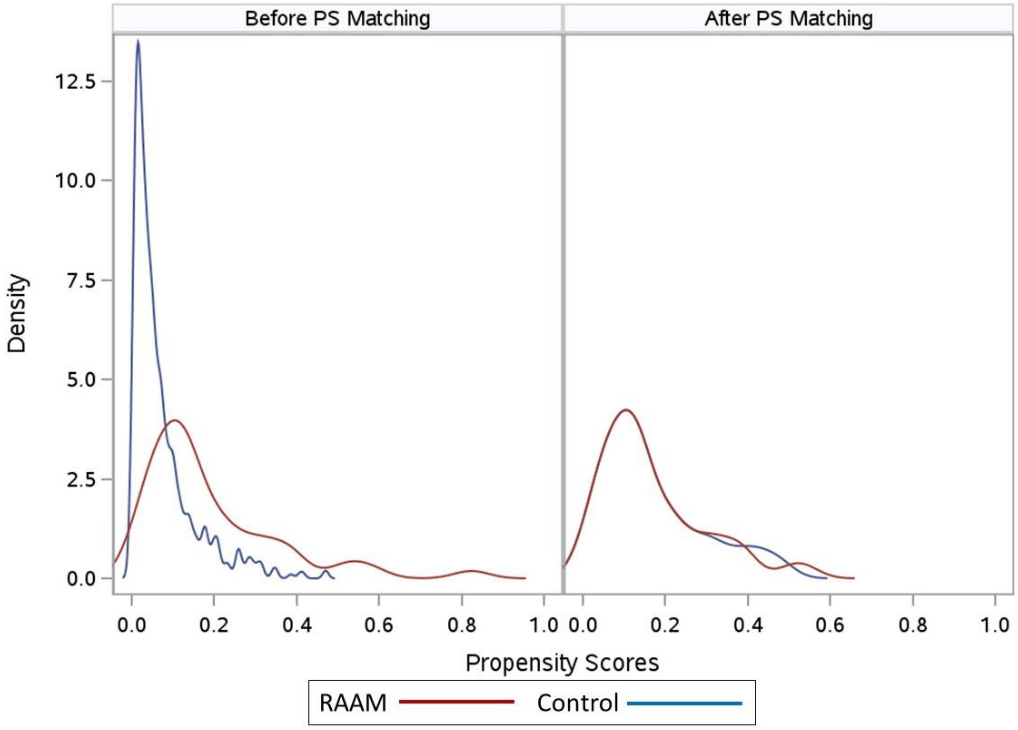

© 2023 Corace K et al. *JAMA Network Open.*
