# Supplementary material for: Rapid Access Addiction Medicine Clinics for People With Problematic Opioid Use
Source: JAMA Netw Open. 2023 Nov 22;6(11):e2344528. doi: 10.1001/jamanetworkopen.2023.44528 (PMC10665968; doi:10.1001/jamanetworkopen.2023.44528)
Supplement: Supplement 2. — Data Sharing Statement [file jamanetwopen-e2344528-s002.pdf]

Data Sharing Statement

Corace. Rapid Access Addiction Medicine Clinics for People with Problematic Opioid Use. *JAMA Netw Open*. Published November 22, 2023. doi:10.1001/jamanetworkopen.2023.44528

Data
Data available: No

Additional Information
Explanation for why data not available: Privacy legislation in Ontario, Canada prevents the sharing of personal health information.
